# Reducing low-value radiological services in Norway –a qualitative multi-professional study on measures and facilitators for change

**DOI:** 10.1186/s12913-022-08077-0

**Published:** 2022-05-21

**Authors:** Eivind Richter Andersen, Bjørn Morten Hofmann, Elin Kjelle

**Affiliations:** 1 Institute for the Health Sciences at the Norwegian University of Science and Technology (NTNU) at Gjøvik, PO Box 1, N-2802 Gjøvik, Norway; 2 Centre for Medical Ethics, University of Oslo, Oslo, Norway

**Keywords:** Low-value imaging, Diagnostic imaging, Health services misuse, de-implementation

## Abstract

**Background:**

Overuse, underuse, and significant variation in the utilisation of radiological services are well documented in the literature. Several radiological examinations are identified as low-value examinations as they do not lead to a change in diagnosis or course of treatment. Even so, such examinations are frequently performed. Many measures for reducing low-value imaging have been carried out with variable outcomes. While there is little evidence as to why some measures work and others do not, adjusting to the context seems important for success. The objective of this study was to investigate which measures stakeholders consider appropriate for reducing the use of low-value imaging and what it takes to make them work.

**Methods:**

Semi-structured interviews were conducted among radiographers, radiologists, radiological department managers, hospital clinicians, general practitioners, and health government/authorities’ representatives. The interview guide covered two broad areas: Experience with low-value services, and possible future measures deemed appropriate for reducing low-value services. Data were analysed in line with a qualitative framework analysis.

**Results:**

The analysis included information from 27 participants. All participants acknowledged that low-value imaging was a problem, but few had very specific suggestions on reducing this in practice. Suggested measures were to stop referrals from being sent, provide support in assessing referrals, or change the healthcare system. Identified facilitators were categorised as management and resources, evidence, and experienced value. In general, appropriate measures should be practical, well-founded, and valuable.

**Conclusions:**

This study provides insight into various stakeholders’ perceptions of suitable interventions to reduce low-value imaging. While many measures for reducing low-value imaging are available, contextual sensitivity is crucial to make them work.

**Supplementary Information:**

The online version contains supplementary material available at 10.1186/s12913-022-08077-0.

## Background

Diagnostic imaging is an essential part of modern patient care and adds value to the clinical evaluation in refining differential diagnoses, decreasing the time required to initiate correct treatment, and contributing to reduced morbidity and mortality [1]. However, radiology is a shared resource across all levels of health care delivery [1]. Using resources on one patient may delay diagnosis and treatment for another patient. Hence, resources should be used as good as possible. There is a new paradigm in health care, to transition from volume-driven to value-driven care, shifting the system from reaction (treating symptoms) to action (preventing care) [2]. Wrong use of radiology may, in fact, be counter-preventive. Sajid and colleagues (2021) state: “if misdiagnosis, mis-referral and delay-to-care are considered patient harm, the number-needed- to-harm (NNH) is only 1.5” [3] Musculoskeletal (MSK)-MRI.

Significant variation in the utilisation of radiological services are well documented, both internationally [4–9] and in Norway [10–12] and may indicate wrong use of radiological services in terms of overuse and underuse. Several radiological examinations are defined in the literature as low-value examinations as they do not lead to change in diagnosis or course of treatment [13, 14]. However, such examinations are still frequently performed [15], giving cause for concern in terms of patient safety, quality of services, and poor priority setting. The lack of interaction between private and public health services, inappropriate referrals, time pressure, and loyalty towards colleagues are examples of drivers of low-value care [10]. Furthermore, the perception of difficulties in referral to a specialist without imaging and fear of disappointing patients have been reported as barriers to reducing the use of low-value imaging [16]. Many measures for reducing low-value imaging (e.g., guidelines, clinical decision support, feedback, health information exchange and education) have been carried out with various outcomes [17]. Measures seem to depend on local culture and health care organizations, but there is little evidence to indicate why some work. However, multimodal measures seem to be most successful [17].

The valuation of specific examinations and measures for reducing low-value imaging may vary between various stakeholders. For example, imaging for lower back pain (LBP) is recognised as an examination that, in general, should not be done without the presence of specific red flags [18]. However, previous studies have shown that patients and professionals have divergent views on the value of low-value imaging and how to reduce the use of these examinations [19, 20].

Changing practice is often considered an individual issue where general practitioners (GPs) need to change their ordering practices and/or patients need more information to make wiser choices [21]. However, multiple factors impact on decisions and behaviour, such as the organisation of health services, norms, regulations, and the economic system [22]. Both outer context determinants (e.g., policy and political support), and inner context determinants (e.g. setting characteristics, organisational culture, work/care processes, interaction between professionals) are found to both facilitate and hinder change [23]. In order to understand this complex system, it is crucial to gain insight into the context from various stakeholders. For the purpose of this study, measures were broadly defined as “the process of identifying and removing harmful, non–cost-effective, or ineffective practices based on tradition and without adequate scientific support” [24] while facilitators were defined as “those elements that make adopting a new behaviour or practice easier” [25]. This study is a part of a larger project with several objectives examined in one data set. The objective of this study was to investigate measures stakeholders consider appropriate for reducing low-value imaging and what it takes to make them work (facilitators).

## Material and method

### Research team and reflexivity

The research team consisted of ERA, a radiographer and PhD-candidate with previous experience of qualitative interviews, AMK (see acknowledgment), a trained medical doctor and PhD-candidate, EK (postdoc) and BMH (professor), both experienced researchers in qualitative methods.

### Study design

This qualitative explorative study was conducted using semi-structured participant interviews. Framework analysis was conducted, consistent with Ritchie and Spencer [26, 27] as methodological orientation to underpin the results. This method is well suited for research that has specific questions, a limited time frame, a pre-designed sample and a priori issues [27].

### Participant selection

Purposive sampling was conducted, using an information letter sent to hospitals and municipal administrations requesting permission to conduct interviews. A contact person helped to identify potential candidates for the study. These candidates were contacted by ERA via email, and written information about the study and a consent form were provided. Only Norwegian speaking candidates consenting to participate in the study was eligible for participation. One participant was approached directly due to specific expertise and experience in the area. Furthermore, two participants were asked directly to participate based on a recommendation from other participants (nested strategy). Candidates willing to participate returned a signed consent form to ERA. The time and place for the interview were then scheduled.

The plan was to include up to 30 participants (5 radiologists, 5 radiographers, 6 managers, 5 hospital clinicians (medical doctors), 5 GPs, and 4 government representatives).

### Setting

The Norwegian healthcare system is mainly divided into 1) Primary health care, organised by the municipalities and consisting of care services, rehabilitation, and social services, 2) Secondary/tertiary healthcare organised by regional health authorities and consisting of hospital trusts and hospitals [28], including most imaging services. All services, including radiology, are mainly public and funded through general taxation [28]. Thus, imaging is basically free of charge. Private imaging centres are also partly commissioned by public health services. However, quicker access to outpatient services is available through out-of-pocket payment or private health insurance policies [28]. Nevertheless, patients need a referral to radiological services. According to the Norwegian radiation protection regulations, referrals shall be based on a patient’s clinical assessment and should contain sufficient information for the healthcare provider to assess the referral [29]. In most cases, this is done by a radiologist.

### Data collection

A semi-structured interview guide was developed, and pilot tested. Interviews were estimated to last 30–60 minutes and the interview guide covered two broad areas: Experience with low-value services, and Possible future measures. The full interview guide is available in Additional file 1. ERA and AMK conducted the interviews as a conversation where participants freely related their experiences and thoughts about the topics. Consequently, the interview guide was not strictly followed. However, ERA and AMK ensured that all questions in the interview guide were covered. Field-notes were not made during the interviews. Due to time limitation and a pragmatic approach, the recruitment ended when an adequate number of various professions was achieved, and the research group agreed, after reading transcriptions, that further interviews most likely would not add additional information.

Due to the COVID-19 pandemic, interviews were performed using video conference (Zoom Video Communications, Inc., San Jose, USA). A formal introduction was used in each interview to ensure that the participants received the same information. Interviews were conducted between February and June 2021, digitally recorded using an external recording device, and subsequently transcribed verbatim.

### Data management and analysis

**Fig. 1:**
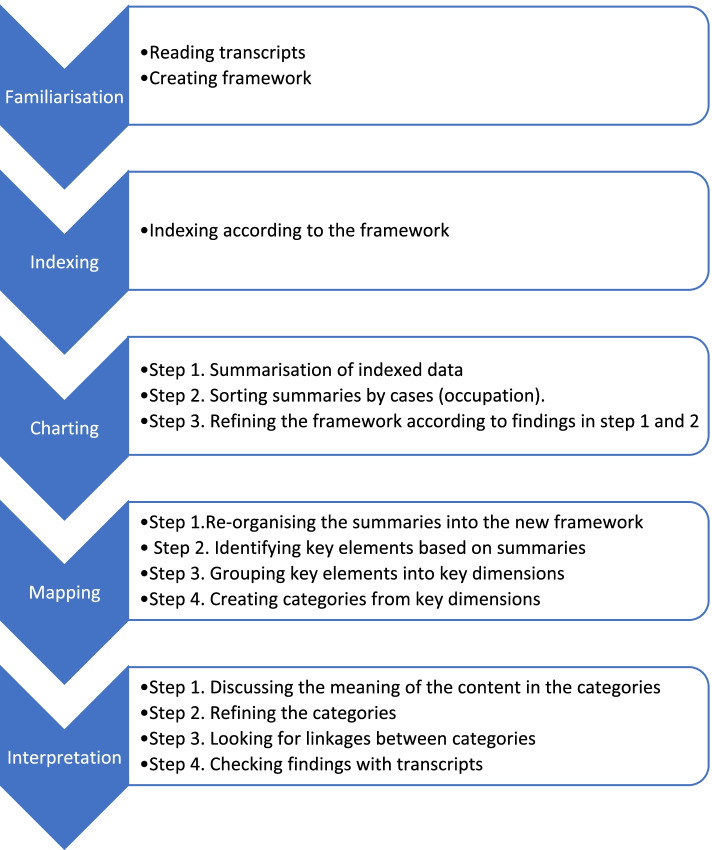
An overview of the analysis process with five main steps

It consists of five main steps: familiarisation, indexing, charting, mapping, and interpretation. An overview of the analysis process is provided in Fig. 1. Each main step was discussed within the research group on several occasions before proceeding to the next stage. A thorough description of the main steps follows below.

#### Familiarisation

ERA and AMK conducted the interviews, and transcription was done by ERA (*n* = 11), AMK (*n* = 7), and EK (*n* = 2), resulting in first-hand knowledge of the material. A professional transcriptionist transcribed the remaining interviews. In the course of one week, EK, AMK, and ERA read two to three transcriptions each day, discussed the content, and identified preliminary codes for the framework. AMK, BMH, and ERA tested and altered the framework, consisting of seven categories and 94 codes, (see additional file 2), by discussing the remaining transcriptions.

#### Indexing

ERA performed the initial indexing according to the framework, using NVivo software: QSR International Pty Ltd. (2021) NVivo (release 1.5.1). Only data relevant to the objective of the current study was indexed. The indexing was discussed and refined by ERA, EK, and BMH.

#### Charting

Summaries were written based on the indexed transcriptions and placed in charts (23). Cases were placed in rows and codes with appurtenant summaries in columns. The summaries adhered to the participants’ own words as closely as possible, interpretations were kept to a minimum, and no material was dismissed as irrelevant at this stage [26]. The framework was revised by sorting, grouping, and rearranging categories and codes based on understanding acquired after reading and discussing the summaries. The new framework consisted of four categories and 13 codes (see additional file 3).

#### Mapping

Mapping and interpretation involve analysis of the critical characteristics in the charts. The summaries were reread, discussed, and reorganised within the new framework. In addition, summaries were sorted by participants’ occupations. Key elements were then identified and grouped in key dimensions which were further categorised. An example of the analysis process from summary to categorization is given in Table 1.

**Table 1:** Example of the analysing process

Summary	Key Elements	Key Dimensions	Categorisation
The government has to say something! Want proper support from the government. They must take the consequences by acknowledging that the gate-keeper function is important. You don’t have a customer relationship in the health services. The government should say so and mean it.	- Should say something - Provide support and take responsibility - Must acknowledge the gate-keeper function	Support from the government	Management
	- No customer relationship in the health services	Personal interaction	Communication

#### Interpretation

The categories were discussed in several rounds, and further analyses were carried out by looking for linkage across categories. The summaries were actively used, providing meaning to the categories. When a consensus of findings was reached, the findings were verified by checking quotations in the transcriptions.

### Ethics

Informed consent was obtained from all participants before the interviews were conducted. The study was approved by the Norwegian Centre for Research Data (NSD), approval number 475812.

## Results

Twenty-seven participants (16 male, 11 female) were included, of whom 17 had more than ten years’ work experience. Demographic details are given in Table 2. The interviews lasted between 37 and 181 minutes (mean 59 minutes).

**Table 2:** Demographic data for the included participants

Workplace	Number of participants
Authorities	4
University hospital	7
Local hospital	9
Medical office/University	2
Medical office	3
Private imaging centre	2
**Geography**	
Urban area	14
Rural area/smaller local hospitals	13
**Occupation**	
*Authorities*	4
*Clinicians*	
General practitioner (GP)	5
Specialist (medicine, orthopaedics, oncology, neurology)	5
*Radiology*	
Radiologist	3
Radiographer	4
Manager	6

All participants acknowledged the problem of low-value imaging and considered the extent of low-value imaging and the challenges it presented to be comprehensive. One radiologist said:

*“It feels like standing under a waterfall trying to stop the water with your bare hands” (p.26).*

**Fig. 2:**
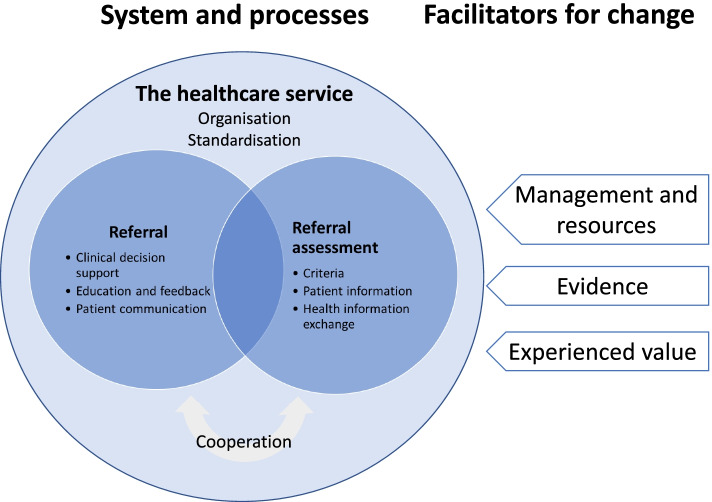
Measures and facilitators for change. Legends: An overview of targets of the measures (referral, referral assessment, and the healthcare service) and facilitators for change. Cooperation was described as essential at both stopping and assessing referrals

The results are presented in two sections: *Measures* and *Facilitators for change*. Participants described several measures having the potential to reduce low-value imaging. Based on initial analysis of the summaries, the measures were organised into three categories by the target for the measures: The healthcare service, Referrals, and Referral assessment. Furthermore, participants described several facilitators for change, categorised as management and resources, evidence, and experienced value. Figure 2 presents an overview of the targets as well as the facilitators for change.

### Measures

Stopping low-value referral was highlighted as the most promising measure, whereas system change was described as potentially highly effective but difficult to achieve. Measures to change the process of referral assessment were considered less effective and difficult to enforce.

### Referrals

Measures aimed at stopping clinicians from sending inappropriate referrals in the first place were considered the most important strategy. One radiologist said:

*“If you get the referral, it’s much harder to stop the examination than if you hadn’t received it [the referral] at all, so to speak. It’s as simple as that.” (p.18).*

Clinical decision support, training courses, and reminders of what constitutes appropriate imaging were suggested as suitable measures. Such measures would increase the clinician’s knowledge about proper imaging and would thus be of support when interacting with patients. Furthermore, the participants mentioned decision support and feedback as suitable since the clinicians, especially GPs, often felt alone in challenging situations with few opportunities to ask for help or guidance. Participants described poor lines of communication between referrers and the radiology department as one of the challenges. This was particularly applicable between radiologists and referrers in primary care. The participants emphasised that successful measures should aim to strengthen the dialogue between health service levels. One clinician stated:

*“A good dialogue with the radiologist is important. It can help you a lot … in imaging with the right indication” (p21).*

Also, radiologists and managers believed that better interaction with clinicians would be helpful. Suggested solutions were using practice coordinators (GPs with a part-time position at hospitals), meetings, and available contact information for the referrers directly.

Furthermore, several participants mentioned that politicians undermined the health service by giving the population a wrong impression about the value of imaging services and undermining health care providers by talking about patients as a consumer, thus leading to unnecessary patient demands. Thus, authorities should support the healthcare providers by not giving the population false expectations. One GP stated:

*“The health services are here to help you, but must identify what is useful for you. The patient can’t just pick and choose whatever [health service] they want. The government should make this clear, mean it, and show that they mean it … by giving more active support to the gate-keeper function” (p.22).*

### Referral assessment

Another critical factor in reducing the use of low-value imaging mentioned by the participants was that radiologists must be able to assess referrals received and prioritise examinations. Referral assessment includes, among other things, indication assessment, choosing the modality best suited for the problem at hand, and selecting an adequate imaging protocol. If the examination is considered inappropriate, the radiologist has the opportunity to refuse the imaging request. However, the radiologists and managers felt that prioritising was difficult. Predefined criteria for the assessment of referrals were suggested as a suitable measure, since it was believed to result in a more consistent assessment. One manager said:

*“I would like for us [radiologists] to be allowed to prioritise even ..., that we could prioritise more strictly!” (p.8).*

Several participants highlighted the fear of stepping on the clinicians’ toes and the lack of clinical information about the patients as a reason for difficulties in prioritising. Interestingly, even though radiologists and managers felt their ability to prioritise was limited, one GP stated that refusal of referrals could be of great value in discussions with patients. It could function as a specialist statement. Hence, such refusals may stop demands for further low-value examinations. One GP said:

*“I would share it [the refusal] with the patient...And say, according to the specialist assessment, it [the examination] will not ... it will not give us any more information” (p.6).*

Other measures mentioned were more clinical information and a tentative diagnosis in the referrals. Moreover, access to previous examinations would be of great value to the radiologists. Thus, information exchange systems were desired, such as a national imaging archive and a standardized referral system.

### The healthcare service

The participants described a need to pay attention to the structure of the healthcare service when attempting to reduce the use of low-value imaging. It was suggested that the entire healthcare service should be one organisation. For example, differentiation between private and public services and separation between primary and specialist services were considered disadvantageous. One manager emphasized:

*“The whole service should be under one umbrella. Because I believe that we could have interacted much better ( …*) *with a common, overarching structure” (p.20).*

Several participants said that the health authorities should aim to standardise the health services through regulations, guidelines, and standardised diagnostic pathways. However, health authority representatives emphasized that they did not have the necessary competencies to develop guidelines themselves. Thus, guidelines and measures to reduce low-value imaging on a system level should be designed and developed in cooperation with experts in the field to ensure good quality and legitimacy in the professional community. The participants regarded guidelines and diagnostic pathways as a double-edged sword. Although they could reduce low-value imaging, they were also considered a driver in some instances. Guidelines must therefore be flexible, clear, and frequently revised. Moreover, inconsistent guidelines were described as problematic. One of the clinicians questioned the guidelines, as the recommendations did not seem to reflect the available evidence, undermining the guideline’s value.

*“The guideline says that there is no evidence of the value [of these examinations] on a population level. However, they still recommend them. It’s quite resource-intensive! So it … it’s strange. It’s hard to understand …*” *(p.8).*

### Facilitators for change

#### Management and resources

Participants stated that the government and managers must take charge both at an overarching level and in the health services to achieve sustainable change. Several participants stated that the government should take responsibility, manage, and provide support. The authorities should support measures and support systems as they can see the big picture and put in place a system where everybody pulls in the same direction. They can set up a coherent health service through organisation and standardisation of the health services. The lack of a coherent system was reported as a barrier for reducing low-value imaging. One GP stated that reducing the use of low-value imaging in one place is of no use if there is an expectation that patients can get the same examination elsewhere.

*“If I know it is possible to get it [the examination] round the next corner... it’s difficult [not to refer it]!” (p.22).*

According to the participants, suitable measures must be systemic. On a healthcare level, focusing on low-value imaging should be a management responsibility, not the responsibility of individual healthcare providers. Management involvement was considered an essential premise for prioritising resources and focusing on low-value imaging, both within and outside the imaging department. One radiologist asked for support in reducing low-value imaging from managers outside the radiology department and stated the following:

*“It’s about … about the management and that ... someone in the clinical environment … someone should try to say “this is not the correct use of radiology””. (p.8).*

The participants emphasised that adequate resources are essential to facilitate change in low-value imaging utilisation. The timeframe was highlighted as an important issue. Change takes time, and measures should be gradually implemented. Thus, management should offer support systems and education/training related to the measures implemented. Furthermore, several participants stressed that the lack of resources for professionals to attend multi-disciplinary meetings, conferences, or educational sessions where they could discuss cases with colleagues was a barrier to reducing low-value imaging. One manager said:

“*…. Obviously it takes …. the time radiologists are using to prepare for these meetings and discussions with colleagues from other fields, this is time (s) he can’t use to report on examinations” (p.4).*

Some measures may require the use of digital tools. For example, digital communication platforms were considered promising as they enable asynchronized communication. However, participants underscored that such platforms should be easy to use and to access as well as being practical and integrated into already existing software. One clinician stated:

*“Because our everyday worklife is busy … You just have to get things done. So, things that contribute to more clicks on the computer. It will not be well-received” (p.15).*

#### Evidence

Participants stated that there must be sufficient support and evidence for selecting the type of low-value imaging to be targeted and the choice of measures used to facilitate change. The referrers must relate to the descriptions of low-value utilisation. According to the GPs, evidence should be from the same context where measures are implemented. As guidelines and proposed changes were often developed by experts in the field, they were most relevant for patients at the specialist healthcare level. If a specific measure was implemented in one practice, participants said that it would be optimal to have data on low-value utilisation from that local practice, not national or regional data. In addition, in order to establish ownership of the new routines, the professional environment and individual professionals should be involved in designing and implementing measures. One clinician said:

*“We [clinicians] would have to agree that it [the examination in question] was unnecessary, or else... we wouldn’t think it [the intervention] was good” (p.1).*

#### Experienced value

To motivate healthcare providers to accept change, participants asserted that measures should address issues the clinicians experienced as problematic rather than theoretical issues. Thus, the clinicians needed to consider the measures implemented as valuable in their day-to-day activity. Furthermore, if there is a personal gain, it will be easier to get healthcare providers on board. For example, meeting and discussing cases with colleagues was considered both sociable and stimulating for GPs. One GP said:

*“All activities where we can meet in a sociable, professional manner, will be well received” (p.22).*

However, the negative consequences for day-to-day activity needed to be kept at a minimum. Implementing a measure in one place may have huge consequences elsewhere. For example, one clinician said that if they had to assess the patient physically before ordering imaging at routine follow-ups, the department’s logistics would be disrupted. Consequently, measures disrupting the workflow would not be well received.

The participants emphasized that attempting to reduce the use of imaging could result in dissatisfied patients and healthcare providers. It could lead to unpleasant situations for the healthcare provider as the relationship with the patient or colleagues could suffer. The individual threshold for accepting negative consequences varies. An essential premise for successful measures was, therefore, sufficient support and security for the individual professional. As one manager stated:

*“You’re quickly distrusted as ‘the difficult one’ if you adopt that role … And you intend to prioritise” (p.7)*.

In addition, participants wanted measures that would help patients understand the problem of low-value imaging, i.e., reducing the patients’ feeling of losing beneficial services. Consequently, measures targeting patients, or the public would be of great support to the referrer as one GP stated:

*“It’s much easier to get the referrer, doctors or whoever it is.. on board if the public are clearly targeted as well” (p.22).*

## Discussion

The purpose of this study was to investigate which measures stakeholders consider appropriate for reducing the use of low-value imaging and what it takes to make them work. We found that all participants acknowledged the problem of low-value imaging. Suggested measures included stopping referrals from being sent, providing support in referral assessment, or changing the healthcare system. However, participants had few specific suggestions on how such measures should be implemented and applied in real life.

Low-value care practices are found in many fields, such as medication prescription, imaging, screening, diagnostic testing, non-surgical and surgical procedures and radiation therapy [23]. It may be argued that health care’s greatest challenge is to identify optimal implementation methods for evidence on a clinician- and system-level [30]. Thus, this study is an important contribution to the field of de-implementation. Furthermore, barriers to implementation and change in clinical practice exist at the patient, provider, departmental and institutional level [30, 31]. Therefore, we consider a multi-professional approach vital when attempting to reduce low-value imaging. To our knowledge, this is the first study investigating suitable measures across all levels of the health service, including governmental level.

### Measures

Measures to stop the referrals from being sent were described as the most promising, for example, by using clinical decision support systems, training courses, and reminders. This is in line with findings from a systematic review highlighting clinical decision support, feedback, or actions required from the referrers as promising strategies [17]. Discussing cases with the radiologist was also suggested as an appropriate measure. However, there is a need for better lines of communication. Furthermore, facilitating the participation of the individual clinician or GP in the process and give adequate support to the individual healthcare provider to induce them to accept measures that stop them from sending referrals is vital. This is in accordance with a study by von Dulmen et al. [32] demonstrating that almost 40% of identified factors for reducing low-value care can be related to the individual healthcare provider, mainly in relation to attitude.

We found a lack of opportunity for prioritisation among radiologists. Fear of stepping on someone’s toes was described as one reason for this. On the other hand, referrers would appreciate rejections, both in order to learn and to use the rejection as support when discussing the value of radiological imaging with patients. Hence, a rejection from the radiologists may act as a support tool for the clinician. In addition, insufficient patient information in the referral made prioritising challenging, and difficulties in collecting additional patient information in the radiology department were described. This is in line with a previous study that reported insufficient referral information as the most frequent cause of unnecessary investigations [33]. We found that measures aimed to increase the quality of referrals, to get easier access to the clinicians, and to make room for prioritisation were preferable.

Our study indicates that healthcare services need to be well organised and have a culture that facilitates coordination and communication within and across healthcare service levels. This is in line with a previous study, describing both different types of culture and lack of communication between professionals as a determinant for the use of low-value care [23]. Changing the healthcare service was perceived to be a suitable but challenging measure to reduce low-value imaging in this study.

Standardisation is one way to manage and change health care services. Thus, implementing guidelines, either alone or combined with other measures, has been reported as the most common intervention evaluated to reduce low-value imaging on a service level [17]. However, our study showed standardisation as a double-edged sword since it may lead to less reflection and professional autonomy, and to reduce the focus on individual-based medicine. Thus, the system must promote standards that can be deviated from without consequences for the individual healthcare provider. Interestingly, a previous study from Norway reported that only 35.7% of clinicians and 45.7% of radiologists used referral guidelines [34]. This suggests that the implementation of guidelines could be improved in the Norwegian healthcare service.

### Facilitators for change

Several facilitators for implementing successful measures were described. However, the facilitators are not a guarantee for success as the context plays a vital part [30].

Management was described as essential as managers can focus on low-value imaging and prioritise resources to implement change. Furthermore, managers can promote a positive culture for de-implementation, for example, by allocating time and resources to radiologists to assess and prioritise referrals and support measures to reduce low-value imaging in all departments so that this could be a coordinated effort. This is in accordance with earlier research that identified facilitators as good leadership and coordination between professionals, mainly at the organisation level [20]. Furthermore, a team approach is essential to creating a positive culture, collaboration and facilitating good communication [32].

This study showed that to achieve change, all levels of health care, from the authorities to the individual health professional, must acknowledge the problem. However, due to contradictory scientific evidence, scepticism is reported as an important barrier for reducing low-value care [20]. de Wit et al. [30] emphasise that physicians must understand the evidence and be willing to change. This finding is elaborated in our study. We found that measures must be knowledge-based, and follow recognised high-quality evidence. Thus, evidence from one’s own workplace, such as feedback or referral statistics, was an important facilitator for getting healthcare providers on board with the measure.

Our study revealed that measures must be experienced as positive in the participants’ day-to-day activity. Measures should be practical, easily accessed, and have few negative consequences on their workday. Furthermore, low-value care must be recognisable and not perceived as theoretical. Earlier research also reports that the healthcare provider must acknowledge a gap in the local practice to come on board with measures [30]. Further, relevant information must be provided to patients as patient knowledge is reported as both a barrier and facilitator for reducing low-value care [23]. Engaging patients has been reported as an effective measure in decreasing the use of low-value care [35]. Traeger et al. [20] found that a leaflet about overdiagnosis could support a delayed prescribing approach to imaging for low back pain. Interestingly, we found that a patient component was important in persuading referrers to agree to measures, as patients would more easily understand why imaging may not be indicated. Thus, such features would work as support to the referrers. This is in accordance with von Dulmen [32], who states that patients and physicians may find it difficult to accept that a care practice that they believed to be effective is not. Therefore, suitable measures must ensure that patients do not feel that a valuable service is taken away to save money.

### Trustworthiness

To achieve trustworthy and transparent reporting, this study is in line with the *consolidating criteria for reporting qualitative research: a 32-item checklist for interviews and focus groups* [36]. Salmon et al. [37] state that the data collection and analysis in qualitative research is highly dependent on the researcher. Therefore, different researchers using the same methods are likely to write different papers [37]. The authors’ contributions, the sampling strategy, and the analysis process have been thoroughly described. In addition, quotations are used to support findings.

In this semi-structured interview study, we included participants across all levels of the health services. One limitation of the study is that non-clinicians with a right to refer imaging, such as manual therapists and chiropractors, were not included. Nor were patients included, resulting in a lack of important perspectives. However, due to the extensive material, the inclusion of more informants could result in poor quality in the data analysis due to a surfeit of data material. Moreover, the recruitment strategy may have led to a selection bias as only participants especially interested in the topic may have been included. As the contact person approach potential participants, we have no record of how many refused to participate. Hence, they may not represent the general view. Nevertheless, we sought informants who provided rich descriptions, yielding insight into the topic [38].

Although the aim was to interview 30 participants, 27 were interviewed in total, and there were fewer radiologists and radiographers compared to the original project plan. However, four of the managers were radiologists, and one was a radiographer. The research team agreed that a sufficient sample size had been achieved in relation to the objective during the familiarisation process. Therefore, inclusion stopped at 27 participants due to pragmatic considerations and a limited time frame.

This study reports on one of several objectives examined in one data set. This resulted in a somewhat less targeted interview guide and a need for modification of the initial framework. However, the framework was mainly used for sorting data, and the modification did not influence the content of the analysis.

All interviews were conducted online, thereby losing the positive aspects of a face-to-face meeting. Some technical difficulties were experienced during some interviews (e.g., poor sound, loss of internet connection). Nevertheless, all interviews were conducted as planned. Digital interviews may have been positive for the participants as they could participate from their workplace. This is in accordance with a previous study, suggesting Zoom as a viable tool for qualitative data collection because of its relative ease of use, cost-effectiveness, data management features and security options [39].

### Further research

Further research should include patients and non-medical referrers as important stakeholders. In addition, subsequent/future research should aim to compare the various stakeholders’ perceptions on suitable measures and assess other contexts.

## Conclusion

The objective of this study was to investigate measures stakeholders consider appropriate for reducing low-value imaging and what it takes to make them work. Hence, this study provides valuable insight into various stakeholders’ perceptions of suitable interventions to reduce low-value imaging. All participants acknowledged the use of low-value imaging as a problem, but few had specific suggestions to reduce this practice. Suggested measures included stopping referrals from being sent, providing support in assessing referrals, or changing the healthcare system. As such, measures may target both an individual and organisational level. Described facilitators for change included management and resources, evidence, and experienced value. However, different stakeholders may experience measures and see opportunities and challenges in multiple ways. Support to the individual and avoidance of unintended consequences were considered vital. Thus, contextual knowledge is crucial to designing and implementing measures and to make them work in order to reduce low-value imaging.

## Supplementary Information

**Additional file 1.**

**Additional file 2.**

**Additional file 3.**

## Data Availability

The datasets generated during and analyzed during the current study are not publicly available due to individual privacy considerations but are available from the corresponding author on reasonable request.

## Abbreviations

GPs: General Practitioners
LBP: Lower back pain
