# Supplementary material for: Reducing low-value radiological services in Norway –a qualitative multi-professional study on measures and facilitators for change
Source: BMC Health Serv Res. 2022 May 21;22:678. doi: 10.1186/s12913-022-08077-0 (PMC9122550; doi:10.1186/s12913-022-08077-0)
Supplement: Supplementary file 1 — Additional file 1. [file 12913_2022_8077_MOESM1_ESM.docx]

Additional file 1. Interview guide

| 1. **Experiences**   What are your reflections on the definition of low-value care? Can you provide some examples of examinations in your practice that will fall under this definition?  Can you tell me about your experience with low-value radiology?  What do you think are the most important reasons for using low-value examinations?  How do you think the following impact on the use of low-value services?   - Economics - Organisational structures   - Systems   - Environmental mechanisms (access to services, demands, expectations)   - Imaging ‘just to be sure’   Have you tried to reduce the use of low-value radiology? If so, what did you do, and how did the measure work?   - How was the measure received? - Why do you think it worked/didn’t work?  1. **Possible future measures**   In your opinion, what does it take to reduce the use of low-value radiology?  What does it take for the measure to work?  What can make measures unsuccessful?  How do you think the measures will be received, for example by:   - 1. Colleagues   2. Patients/next of kin   3. Others  1. **Elaboration**   Is there anything else you think it is important for us to know or that you wish to elaborate? |
| --- |
