# Supplementary material for: Reducing low-value radiological services in Norway –a qualitative multi-professional study on measures and facilitators for change
Source: BMC Health Serv Res. 2022 May 21;22:678. doi: 10.1186/s12913-022-08077-0 (PMC9122550; doi:10.1186/s12913-022-08077-0)
Supplement: Supplementary file 2 — Additional file 2. [file 12913_2022_8077_MOESM2_ESM.docx]

Additional file 2. Framework

| 1.0 | **Health policy and organisation** |
| --- | --- |
| 1.1 | Politics |
| 1.2 | Laws and regulations |
| 1.3 | Government |
| 1.4 | Social economics |
| 1.5 | Organisation of healthcare services |
| 1.6 | Access to services |
| 1.7 | Capacity and prioritising |
| 1.8 | Resources |
| 1.9 | Logistics |
| 1.10 | Financial incentives |
| 1.11 | Guidelines and patient pathways |
| 1.12 | Routines |
| 1.13 | Roles and tasks |
| 1.14 | Time pressure |
| 1.15 | Patient economics |
| 1.16 | Justice |
| 1.17 | Dignity |
| 1.18 | Management |
| **2.0** | **Quality in health care services** |
| 2.1 | Value for patients and next of kin |
| 2.2 | Value for referrers |
| 2.3 | Value for society |
| 2.4 | Estimation of benefits vs. costs |
| 2.5 | Referral quality |
| 2.6 | Efficiency vs. quality |
| 2.7 | Risks |
| 2.8 | Quality indicators |
| **3.0** | **Knowledge and competence** |
| 3.1 | Knowledge |
| 3.2 | Competence |
| 3.3 | Lack of competence |
| 3.4 | Research |
| 3.5 | Evidence |
| 3.6 | Opportunity to stay up to date |
| 3.7 | Access to information |
| **4.0** | **Interaction and communication** |
| 4.1 | Dialogue between patient and referrer |
| 4.2 | Dialogue between hospital referrers and radiologists |
| 4.3 | Dialogue between GPs and radiologists |
| 4.4 | Trust |
| 4.5 | Co-selection |
| 4.3 | Interaction |
| 4.4 | IT-systems |
| **5.0** | **Attitude and culture** |
| 5.1 | Prioritisation challenges |
| 5.2 | Expectations to the radiological department |
| 5.3 | Defensive medicine |
| 5.4 | Management of uncertainties |
| 5.5 | Examination hierarchy |
| 5.6 | Professional ambition |
| 5.7 | Market-driven thinking |
| 5.8 | Expectations and desires |
| 5.9 | Resignation |
| 5.10 | Awareness |
| 5.11 | Willingness to change |
| 5.12 | Discretionarily assessments |
| 5.13 | Healthcare professionals’ authority |
| **6.0** | **Measures for reducing low-value imaging** |
| 6.1 | Consciousness-raising |
| 6.2 | Better interaction |
| 6.3 | Better communication |
| 6.4 | Guidelines and patient pathways |
| 6.5 | Decision support |
| 6.6 | Internal invoicing |
| 6.7 | Dedicated imaging slots |
| 6.8 | Revisions |
| 6.9 | Common IT-systems |
| 6.10 | Referral assessment |
| 6.11 | Feedback/guidance |
| 6.12 | Rejections |
| 6.13 | Education and courses |
| 6.14 | Discussion groups |
| 6.15 | Await/make plans |
| 6.16 | Change the financial system |
| 6.17 | Change in management |
| 6.18 | Right to order imaging |
| 6.19 | Multimodal measures |
| **7.0** | **Measure characteristics /effects** |
| 7.1 | Premise for measures |
| 7.2 | Implementation |
| 7.3 | Considerations |
| 7.4 | Experiences and assessment |
