# Supplementary material for: Reducing low-value radiological services in Norway –a qualitative multi-professional study on measures and facilitators for change
Source: BMC Health Serv Res. 2022 May 21;22:678. doi: 10.1186/s12913-022-08077-0 (PMC9122550; doi:10.1186/s12913-022-08077-0)
Supplement: Supplementary file 3 — Additional file 3. [file 12913_2022_8077_MOESM3_ESM.docx]

Additional file 3. Revised framework.

| **1.0** | **Health policy and organisation** |
| --- | --- |
| 1.1 | Government/health authorities |
| 1.2 | Organisation of the healthcare services |
| 1.3 | Prioritising |
| 1.4 | Resources and time |
| 1.5 | Guidelines and procedures |
| 1.6 | Opportunity for change |
| **2.0** | **Knowledge and evidence** |
| 2.1 | Evidence |
| **3.0** | **Interaction and communication** |
| 3.1 | Dialogue between patient and referrer |
| 3.2 | Dialogue between hospital referrers and radiologists |
| 3.3 | Interaction |
| 3.4 | IT-systems |
| **4.0** | **Attitude and culture** |
| 4.1 | Awareness |
| 4.2 | Willingness to change |
